# Current status of Kampo medicine curricula in all Japanese medical schools

**DOI:** 10.1186/1472-6882-12-207

**Published:** 2012-11-02

**Authors:** Makoto Arai, Shuichi Katai, Shin-ichi Muramatsu, Takao Namiki, Toshihiko Hanawa, Shun-ichiro Izumi

**Affiliations:** 1 Department of Oriental Medicine, Tokai University School of Medicine, 143 Shimokasuya, Isehara, Kanagawa, 259-1193, Japan; 2 Faculty of Health Sciences, Tsukuba University of Technology, 4-12-7 Kasuga, Tsukuba, Ibaraki, 305-8521, Japan; 3 Division of Oriental Medicine, Center for Community Medicine, Jichi Medical University, 3311-1 Yakushiji, Shimotsuke, Tochigi, 329-0498, Japan; 4 Department of Japanese-Oriental (Kampo) Medicine, Graduate School of Medicine, Chiba University, 1-8-1 Inohana, Chuo-ku, Chiba, 260-8670, Japan; 5 Oriental Medicine Research Center, Kitasato University, 5-9-1 Shirokane, Minato-ku, Tokyo, 108-8641, Japan; 6 Department of Academic and Student Services, Tokai University School of Medicine, 143 Shimokasuya, Isehara, Kanagawa, 259-1193, Japan

**Keywords:** Kampo medicine, Traditional Japanese medicine, Education, Medical school, Curriculum standardization, Questionnaire survey

## Abstract

**Background:**

There have been a few but not precise surveys of the current status of traditional Japanese Kampo education at medical schools in Japan. Our aim was to identify problems and suggest solutions for a standardized Kampo educational model for all medical schools throughout Japan.

**Methods:**

We surveyed all 80 medical schools in Japan regarding eight items related to teaching or studying Kampo medicine: (1) the number of class meetings, target school year(s), and type of classes; (2) presence or absence of full-time instructors; (3) curricula contents; (4) textbooks in use; (5) desire for standardized textbooks; (6) faculty development programmes; (7) course contents; and (8) problems to be solved to promote Kampo education. We conducted descriptive analyses without statistics.

**Results:**

Eighty questionnaires were collected (100%). (1) There were 0 to 25 Kampo class meetings during the 6 years of medical school. At least one Kampo class was conducted at 98% of the schools, ≥4 at 84%, ≥8 at 44%, and ≥16 at 5%. Distribution of classes was 19% and 57% for third- and fourth-year students, respectively. (2) Only 29% of schools employed full-time Kampo medicine instructors. (3) Medicine was taught on the basis of traditional Japanese Kampo medicine by 81% of the schools, Chinese medicine by 19%, and Western medicine by 20%. (4) Textbooks were used by 24%. (5) Seventy-four percent considered using standardized textbooks. (6) Thirty-three percent provided faculty development programmes. (7) Regarding course contents, “characteristics” was selected by 94%, “basic concepts” by 84%, and evidence-based medicine by 64%. (8) Among the problems to be solved promptly, curriculum standardization was selected by 63%, preparation of simple textbooks by 51%, and fostering instructors responsible for Kampo education by 65%.

**Conclusions:**

Japanese medical schools only offer students a short time to study Kampo medicine, and the impetus to include Kampo medicine in their curricula varies among schools. Future Kampo education at medical schools requires solving several problems, including curriculum standardization.

## Background

Kampo medicine, or traditional Japanese medicine, generally includes not only “Kampo-yaku” (herbal medicine) but also massage, moxibustion, acupuncture, and acupressure [1,2]. The word “Kampo” is a Japanese word meaning “Chinese way” reflecting its origin in China. Since its introduction into Japan from China 1500 years ago, Kampo medicine has greatly developed the way of diagnosis, i.e., “the abdominal examination”, by which the type of Kampo medicine is selected and was practiced in Japan as the primary type of medicine [2]. However, the medical system reform conducted in the 19th century under direction of the Meiji government adopted new curricula of medical education based on Western medicine, resulting in the elimination of Kampo education from the Japanese medical schools’ curricula [3,4].

In recent years, an increasing number of people, even in western countries, have been using complementary and alternative medicine (CAM) including Kampo medicine [5], and in parallel there has been a steady increase in the number of medical schools that have added CAM therapies to their curricula [6]. The Japanese Ministry of Education, Culture, Sports, Science and Technology (MEXT) announced in 2001 that instruction of basic Kampo medicine was to be incorporated into the core curricula of all medical schools. Since then, among the 80 Japanese universities with medical schools, a rapidly increasing number of them have integrated Kampo medicine into their curricula [7]. However, regarding the current status of Kampo education in medical courses in Japan, to our knowledge, only a few reports provide basic information on the spread of Kampo education with details on the curricula. The aim of the present study was, therefore, to survey the current status of Kampo education at all the medical schools in Japan and to identify some problems and suggest solutions to implement a standardized Kampo educational system.

## Methods

In July 2011, we conducted a nationwide postal questionnaire survey of all 80 Japanese medical schools (51 national and public universities and 29 private universities). The persons actually responsible for Kampo education in each of the universities were asked to respond to the questions in consultation with the medical curriculum administrator and to declare the names of their universities and the responders. For a precise survey, additional surveys by phone or post were carried out for the universities that did not respond timely or responded inadequately. The questionnaire consisted of eight items specifically related to teaching or studying Kampo medicine, unless otherwise stated: (1) the number of class meetings (taught sessions), target school year or years, and type of classes; (2) presence or absence of a full-time instructor or instructors; (3) curricula contents; (4) textbooks in use; (5) desire for standardized textbooks; (6) faculty development programmes; (7) course contents; and (8) problems to be solved to promote Kampo education (see Additional file 1). When we analyzed the accumulated data, we counted one class meeting as one in which 50% or more of the content was regarding Kampo medicine. Cases of laboratory assignments in the third or fourth year and clinical clerkships in the fifth and sixth years were excluded as class meetings.

The study was funded by a grant from the Ministry of Health, Labour and Welfare. The survey was approved by the Institutional Review Board for Clinical Research of Tokai University and conformed to the principles of the Helsinki Declaration. To prepare the questionnaire, we modified questions from a questionnaire from a similar study conducted by the Liaison Committee of The Japan Society for Oriental Medicine in 2007 [8], after obtaining the author’s permission. This study was a survey of the current status of Kampo education in all 80 Japanese medical universities and was not intended to include statistical analyses. The appropriate responsible persons from all 80 medical schools gave written informed consent to participate in this study.

## Results

### Study population

A total of 80 questionnaires were collected, which means that responses were obtained from each of the 80 medical schools in Japan (response rate, 100%).

### Number and length of class meetings and target school year or years

**Figure 1:**
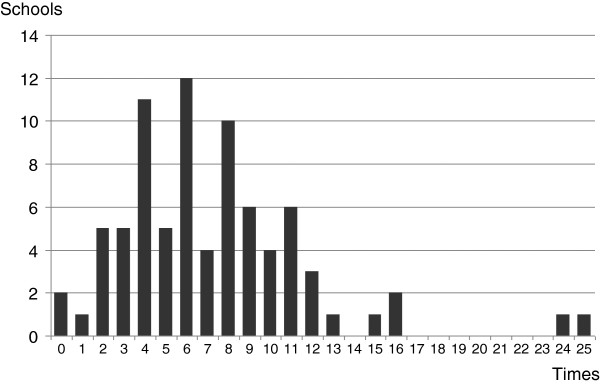
**The number of required Kampo class meetings in all 80 medical schools.** Mean, 7.25 ± 4.45 times; Median, 6 times; ≥1, 78/80 schools (98%); ≥4, 67/80 schools (84%); ≥8, 35/80 schools (44%); ≥16, 4/80 schools (5%).

**Figure 2:**
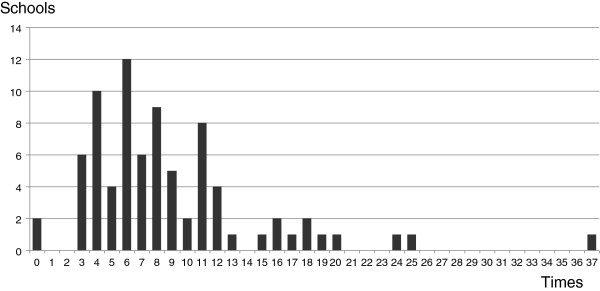
**The number of required and elective Kampo class meetings in all 80 medical schools.** Mean, 8.79 ± 5.87 times; Median, 7.5 times; ≥1, 78/80 schools (98%); ≥4, 72/80 schools (90%); ≥8, 40/80 schools (50%); ≥16, 10/80 schools (13%).

**Figure 3:**
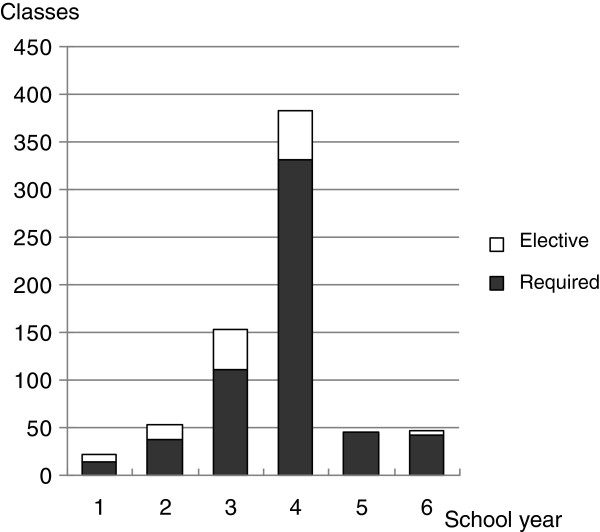
The number of Kampo class meetings by school year in all 80 medical schools.

The length of one class meeting ranged from 45 to 100 minutes, with a mean of 80.5 ± 13.6 minutes and a median of 90 minutes. The number of Kampo class meetings as a required subject during the 6 years of medical school ranged from 0 to 25 (at 2 and 1 school, respectively), with a mean of 7.25 ± 4.45 class meetings and a median of 6 class meetings (Figure 1). The total number of required and elective Kampo class meetings ranged from 0 to 37 (at 2 and 1 school, respectively) with a mean of 8.79 ± 5.87 class meetings and a median of 7.5 class meetings (Figure 2). At least 1 Kampo class meeting was conducted as a required subject at 78 medical schools (98%), 4 or more at 67 schools (84%), 8 or more at 35 schools (44%), and 16 or more at 4 schools (5%). Regarding elective classes in addition to these required classes, at least 1 class meeting was taught at 78 schools (98%), 4 or more at 72 schools (90%), 8 or more at 40 schools (50%), and 16 or more at 10 schools (13%). Distribution of the number of Kampo class meetings as a required subject by school year was: 2%, 7%, 19%, 57%, 8%, and 7% for first- through sixth-year students, respectively (Figure 3). Practical training in required Kampo classes was given at 12 schools (15%), and combined required or elective classes at 17 schools (21%). Required clinical clerkships were offered at 10 schools (13%) and required or elective clinical clerkships at 21 schools (26%). Kampo laboratory could be selected in a few-months-long laboratory assignment programmes for third- or fourth-year students at only 2 schools (2.5%).

### Educational systems and contents

**Figure 4:**
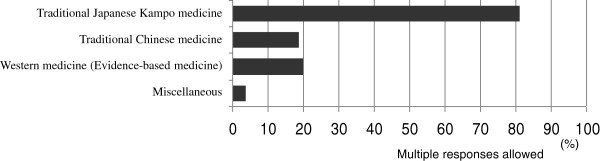
Suggested curriculum contents for teaching traditional Japanese (Kampo) medicine at all 80 medical schools.

**Figure 5:**
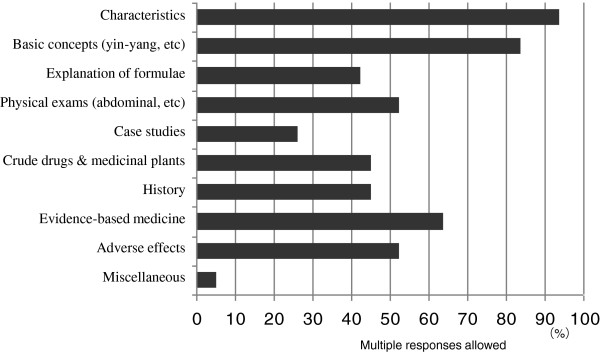
The contents of Kampo education in 6 years of medical school.

Of the 80 medical schools, 23 schools (29%) employed full-time instructors to teach traditional Japanese Kampo medicine, while 57 schools (71%) did not have any such instructors. Teaching was based on traditional Japanese Kampo medicine at 65 schools (81%), traditional Chinese herbal medicine at 15 schools (19%), Western medicine (evidence-based medicine [EBM]) at 16 schools (20%), and “miscellaneous” at 3 schools (4%) (Figure 4). Miscellaneous answers included history, pharmacology, and diversity of medical treatment at 1 school each with multiple responses being allowed. A textbook was used at 19 schools (24%), while no textbook was used at 61 schools (76%). However, 59 schools (74%) wanted to consider the use of standard textbooks, if any were available, 16 schools (20%) were not considering using textbooks, and 5 schools (6%) did not respond. Faculty development programmes were conducted in 26 schools (33%) versus 54 schools (67%) that did not. Regarding the contents to be taught before graduation, characteristics (distinctive features of Kampo medicine, differences between Kampo and Western medicine, etc.) was selected by 94% of the schools, basic concepts (yin and yang, deficiency and excess, etc.) by 84%, explanation of formulae by 43%, practical training for physical examinations (including abdominal, pulse, and tongue examinations) by 53%, case studies by 26%, crude drugs and medicinal plants by 45%, history by 45%, EBM by 64%, adverse effects (adverse drug reactions) by 53%, and miscellaneous by 5% (Figure 5). Miscellaneous answers included current status of medical treatment by 3%, clinical topics by 1%, and attitude as a clinician by 1% with multiple responses being allowed.

### Problems

**Figure 6:**
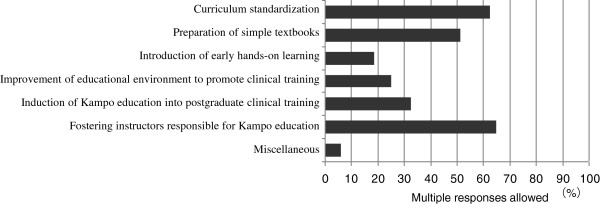
Problems to be solved promptly toward standardization of Kampo education.

Among the problems to be solved promptly in the area of Kampo education, curriculum standardization was selected by 63% of the schools, preparation of simple textbooks by 51%, early hands-on learning by 19%, improvement of the Kampo educational environment to promote participatory clinical training by 25%, introduction of Kampo education into both early and late postgraduate clinical training by 33%, fostering instructors responsible for Kampo education by 65%, and miscellaneous by 6% (Figure 6). Miscellaneous answers included the removal of prejudice against Kampo medicine by 3%, and establishing questions for CBT (Computer-based Testing) and the National Medical Licensing Examination (NMLE) by 3%. These data were acquired with multiple responses being allowed.

## Discussion

Regarding the status of Kampo education at Japanese medical schools, a nationwide phone survey was conducted in 1998 before incorporation of Kampo medicine into the core medical curricula, reporting that only 18 of the 80 Japanese medical schools had either elective or required classes on Kampo medicine [4]. Thereafter, several survey reports were published on the recent state of Kampo education offered at many medical schools [8,9]. These surveys were, however, incomplete and unsatisfactory because the methods and criteria of the surveys were unclear [8], and not all of the medical schools responded to the questionnaires (response rate, 84%) [9]. In the 10 years since 2001, when Kampo medicine was first integrated into the core medical curricula, the present study is the first to have retrieved questionnaires from all 80 of the medical schools in Japan (response rate, 100%).

In both China and South Korea, education in traditional medicine is offered at different universities from those that focus on modern Western medicine, and the requirements for qualification as a medical doctor also differ. The formal medical education at universities of traditional Chinese herbal medicine in China requires 5 years of instruction, and students study traditional Chinese medicine along with modern Western medicine in curricula with ratios of each type of medicine from 7:3 to 6:4, respectively [10-12]. Even in schools in China that focus on Western medicine, around 80 class hours of traditional Chinese medicine are provided [13]. In Japan, there is only one qualification required to become a medical doctor, and that is to pass the NMLE after studying Western medicine at medical schools. Any Japanese licensed physician can prescribe Kampo formulations [14]. Therefore, 70% to 97% of Japanese physicians prescribe Kampo medicine according to clinical evidence and mechanism of action or by utilizing simple handbooks from the perspective of modern Western medicine [14-16]. In addition, more extensive scientific evidence of Kampo formulae has been accumulated [17,18], and their quality and safety have been maintained at higher levels with the progress of Kampo extract formulations [19], resulting in their substantial integration into Western medicine [20,21]. Nevertheless, Kampo medicine is primarily characterized by unique concepts of traditional medicine, such as “*sho* (pattern),” diagnostic skill, for example the abdominal examination, and therapeutic modalities [22]. Although it takes many hours of classes and hands-on experience to learn these concepts and modalities, there are no universities specializing in traditional Japanese Kampo medicine in Japan like those in China and South Korea. Moreover, Japanese medical schools only offer students traditional Japanese Kampo medicine education for an extremely limited amount of time. In our opinion, sufficient practical Kampo education based on traditional methods should be provided yearly before attempting any clinical use of Kampo formulae as prospective remedies.

A curriculum for medical students consisting of three different courses (the option of a 110-minute class: 4, 8, or 16 times) for Kampo education has been proposed [23]. Figure 1 shows the numbers of required classes, indicating the impetus of each school to promote Kampo education; while Figure 2 shows the total numbers of both required and elective classes, which is important to know in regard to the number of classes available to students who want to learn Kampo medicine. The present survey revealed that the number of required Kampo class meetings was 4 times or more at 84% of schools, but 8 times or more at less than half of the schools (44%), and 16 times or more at only 4 schools (5%). Although the most Kampo class meetings available to students was 25 times at 1 school, there were 2 schools that did not offer any Kampo classes. Obviously, the impetus to include Kampo medicine in curricula varies widely among universities.

Because the class hours of Kampo education are so limited, providing hands-on learning of Kampo techniques and their effects could improve students’ motivation [24]. However, the actual incorporation into the curriculum of such hands-on practice and clinical training (including clinical clerkships) was reported by less than 20% of Japanese medical schools. Because so many well-trained instructors are needed for the implementation of effective Kampo education, the small number of universities employing full-time instructors responsible for teaching Kampo medicine (29%) seems to greatly hinder the improvement of Kampo education [7]. In the present survey as well, fostering instructors responsible for Kampo education was selected by the largest number of responder schools (65%) as a problem to be dealt with promptly. Moreover, conducting faculty development programmes and the use of textbooks were only reported by 33% and 24%, respectively, of the 80 universities surveyed. These results suggested that only several medical schools in Japan had a good educational environment for Kampo medicine. Major factors that delay the development of Kampo education may include the fact that students must spend many hours preparing for the NMLE, which requires the accumulation of a large body of knowledge regarding Western medicine, while questions on Kampo medicine have never been asked on the NMLE [25]. The amount of time spent in educating a certain subject is a reflection of the portion of the NMLE questions on that particular subject.

Contrarily, almost all students are interested in Kampo medicine, and feel it necessary to have opportunities to learn Kampo medicine even after graduation [25]. Actually, almost all physicians involved in community health care use Kampo formulae to some extent. Most of them have not had the benefit of taking any classes in their regular medical education on Kampo medicine, but have learned Kampo medicine by self-study [16]. Moreover, Kampo therapy has become increasingly popular among a great number of Japanese [26-28]. Nevertheless, Japan’s Kampo education programmes do not fully meet the needs or desires of medical students nor those of physicians from a clinical aspect [16,25].

Kampo classes are offered most frequently for 3rd- and 4th-year students (76%). In our opinion, students are expected to gain diverse views of medical treatment and health care by learning about Kampo medicine along with systematically studying Western medicine in their preclinical years. During their internship, they can be engaged in clinical training on patients with perspectives of both Western and Kampo medicine. In the future Japan, a new type of medical care will be developed that comprises Kampo medicine integrated into Western medicine [29]. Now is a very good time to promote Kampo education to encourage the fostering of physicians able to practice Kampo and Western integrative medicine.

Regarding teaching bases of traditional medicine, more than 80% of Japanese medical schools teach Kampo medicine. As the course content, “characteristics” and “basic concepts” of Kampo medicine were selected by 94% and 84% of the responding medical schools, respectively. Kampo medicine is considered to be a body of wisdom cultivated by the long period of Japanese history and culture [29]. Therefore, many leading physicians responsible for Kampo education may recognize the importance of Kampo medicine as medical practice based on a different medical system from that of Western medicine. Because no national standardized programmes for Kampo education are currently available in Japan [2], traditional Chinese herbal medicine is chiefly being taught instead at 19% of the Japanese medical schools. As pointed out by many educators who participated in the present survey, in order to popularize and spread the use of traditional Japanese Kampo medicine, standardization of educational curricula and the preparation of simple textbooks are greatly desired and will be necessary.

A limitation of this study is that the survey only targeted the 2011 curricula at each medical school, which proves to be a selection bias, because the curricula on Kampo medicine have varied from year to year. In point of fact, of the two universities that reported offering no Kampo classes, one is now on the road to a new curriculum in which Kampo education is scheduled for all the medical students by the time they graduate; while at the other, although the medical curriculum does not include Kampo education, it is offered in liberal arts and science courses. For an accurate assessment of the current status of Kampo education, it will be necessary to analyze the current status of each university and to discover trends through repeated follow-up surveys.

## Conclusions

In Japan, traditional medicine (Kampo medicine) is incorporated into the medical curriculum at 98% of the 80 medical schools nationwide, and Kampo class meetings are provided 4 times or more by the time of graduation at 84% of them. However, the impetus to include Kampo medicine in their curricula varies widely. The future establishment of Kampo education at Japanese medical schools requires fostering instructors knowledgeable in and responsible for Kampo education, curriculum standardization, and preparation of simple textbooks.

## Competing interests

We have no competing financial or non-financial interests in this study, however, the Department of Oriental Medicine, Tokai University School of Medicine, and the Division of Oriental Medicine, Jichi Medical University each received a grant from Tsumura, a manufacturer of Kampo medicine in Japan.

## Authors’ contributions

MA and SK conceived the study. MA wrote the manuscript. MA and SK participated in the data collection, data analysis and interpretation of data. SM, TN, TH, and SI carefully revised the manuscript. All authors read and approved the final manuscript.

## Pre-publication history

The pre-publication history for this paper can be accessed here:

http://www.biomedcentral.com/1472-6882/12/207/prepub

## Supplementary Material

Additional file 1 Questionnaire on Kampo Education in the Curricula of Japanese Medical Schools. Click here for file
