# Supplementary material for: Current status of Kampo medicine curricula in all Japanese medical schools
Source: BMC Complement Altern Med. 2012 Nov 2;12:207. doi: 10.1186/1472-6882-12-207 (PMC3528449; doi:10.1186/1472-6882-12-207)
Supplement: Additional file 1 — Questionnaire on Kampo Education in the Curricula of Japanese Medical Schools. [file 1472-6882-12-207-S1.pdf]

Questionnaire on Kampo Education in the Curricula of Japanese Medical Schools

1. In what year or years and how many classes do you actually offer Kampo education to your students? Please mark 'required' or 'elective', and briefly describe the class content (lecture, medical interview and physical examination practice [MIPEP], clinical clerkship, etc.) If additional space is required, please comment freely on an attachment.)

	School year	Required / Elective	Number of classes	Contents (briefly)
(Ex)	3	(R) / E	6	Lecture
	4	R / (E)	2	MIPEP
		R / E		
		R / E		
		R / E		
		R / E		

Total:__________ times

(Required:________ times, Elective:________ times)

2. Are there any full-time instructors for Kampo medicine employed by the university?

   1 YES

   2 NO

3. In what context in your curriculum is Oriental medicine principally taught?

   1 Traditional Japanese Kampo medicine

   2 Traditional Chinese herbal medicine

   3 Western medicine (evidence-based medicine)

   4 Other (specify:______________________________)

4. Are you using any textbooks in your Kampo medicine classes?

   1 YES

   2 NO

   → If YES, please give the titles.

____

(Continued on the back)

5. If any standard Kampo textbooks are available, would you be interested in using them in your classes?

    1 YES

    2 NO

6. Do you have workshops or programmes for faculty development?

    1 YES

    2 NO

7. What do you think should mainly be taught in Kampo education for medical students? (multiple responses allowed)

    1 Characteristics (differences between Kampo and Western medicine, etc.)

    2 Basic concepts (yin and yang, deficiency and excess, etc.)

    3 Explanation of formulae

    4 Physical examination practice (abdominal, pulse, and tongue exams, etc.)

    5 Case studies

    6 Crude drugs and medicinal plants

    7 History

    8 Evidence-based medicine

    9 Adverse effects

    10 Other

    (Specify:____)

8. What do you think should promptly be solved in the area of Kampo education? (multiple responses allowed)

    1 Curriculum standardization

    2 Preparation of simple textbooks

    3 Introduction of early hands-on learning

    4 Improvement of educational environment to promote participatory clinical training

    5 Induction of Kampo education into early and late postgraduate clinical training

    6 Fostering instructors responsible for Kampo education

    7 Other (Specify:____)

*Regarding the content of questionnaire responses, we may contact you.
To help us do so, please write your name and affiliation below.

Name:______________________________ Affiliation:____________________
